# Vitamin D and idiopathic pulmonary fibrosis: a two-sample mendelian randomization study

**DOI:** 10.1186/s12890-023-02589-z

**Published:** 2023-08-23

**Authors:** Tong Lin, Fen Zhou, Haiyan Mao, Zhenye Xie, Yuhong Jin

**Affiliations:** Department of Critical Care Medicine, Ningbo Medical Center Lihuili Hospital, NO.57 Xingning Road, Ningbo, China

**Keywords:** Vitamin D, Idiopathic pulmonary fibrosis, Mendelian randomization

## Abstract

**Background:**

A prospective study of multiple small samples found that idiopathic pulmonary fibrosis (IPF) is often accompanied by a deficiency in Vitamin D levels. However, the causal relationship between the two remains to be determined. Therefore, our study aims to investigate the causal effect of serum 1,25-hydroxyvitamin D (25(OH)D) on the risk of IPF through a two-sample Mendelian randomization (MR) analysis.

**Methods:**

Through data analysis from two European ancestry-based genome-wide association studies (GWAS), including 401,460 individuals for 25(OH)D levels and 1028 individuals for IPF, we primarily employed inverse-variance weighted (IVW) to assess the causal effect of 25(OH)D levels on IPF risk. MR-Egger regression test was used to determine pleiotropy, and Cochran’s Q test was conducted for heterogeneity testing. Leave-one-out analysis was conducted to examine the robustness of the results.

**Results:**

158 SNPs related to serum 25(OH)D were used as instrumental variables (IVs). The MR analyses revealed no evidence supporting a causal association between the level of circulating 25(OH)D and the risk of IPF. The IVW method [OR 0.891, 95%CI (0.523–1.518), *P* = 0.670]; There was no significant level of heterogeneity, pleiotropy and bias in IVs. Cochran’s Q test for heterogeneity (MR Egger *P* = 0.081; IVW *P* = 0.089); MR-Egger regression for pleiotropy (*P* = 0.774).

**Conclusions:**

This MR Study suggests that genetically predicted circulating vitamin D concentrations in the general population are not causally related to IPF.

**Supplementary Information:**

The online version contains supplementary material available at 10.1186/s12890-023-02589-z.

## Background

Idiopathic pulmonary fibrosis (IPF) is a chronic lung disease characterized by progressive pulmonary fibrosis of unknown etiology and reduced pulmonary function [1]. IPF is a relatively rare and a challenging disease to manage due to unpredictable, difficulties in early diagnosis, deficient in median survival period from diagnosis; however, its incidence is increasing in Western developed countries, with an estimated incidence of 2.8–18 cases per million per year [2]. The current FDA-approved drugs (Pirfenidone, Nintedanib) for the treatment of IPF have not been shown in clinical studies to prevent or reverse the progression of IPF or reduce mortality [3, 4]. Therefore, early identification of potential risk factors can help prevent the onset of IPF.

Vitamin D can be obtained through dietary supplementation or synthesized in the human skin following exposure to sunlight. The active hormonal form (1, 25-hydroxyvitamin D) is synthesized in the liver and kidneys. It has been increasingly recognized that Vitamin D plays a role in physiological and pathological processes of the body beyond bone metabolism, including host defense, inflammation, immunity, and repair[5, 6]. Several epidemiological studies have shown that patients with risk of respiratory diseases often have serum Vitamin D deficiency. In patients with COPD, low levels of 25-OHD were significantly correlated with reduced forced expiratory volume in 1 s (FEV1), with more severe COPD stages exhibiting higher prevalence of 25-OHD deficiency [7]. A randomized controlled trial revealed that vitamin D deficiency is common in patients with cystic fibrosis (CF), and high-dose vitamin D therapy can help regulate the inflammatory response of CF by reducing levels of the inflammatory cytokines TNF-a and IL-6 [8]. Furthermore, several small-scale prospective studies investigating the relationship between vitamin D and idiopathic pulmonary fibrosis (IPF) have shown that plasma levels of Vitamin D are generally lower or deficient in IPF patients, and are closely associated with acute exacerbations of IPF [9, 10]. Recently, Tzilas et, al have investigated the potential role of vitamin D in pulmonary fibrosis using animal models and human assessment of plasma Vitamin D levels. The findings suggest that Vitamin D supplementation can prevent lung fibrosis in mice and decrease fibrotic responses in lung fibroblasts. Moreover, Vitamin D deficiency is associated with disease severity and poor prognosis in patients with IPF, indicating its potential as a prognostic biomarker and therapeutic target [11]. However, currently there is no large-scale randomized controlled trial (RCT) to study the relationship between vitamin D and IPF. Only a limited number of prospective studies with small sample sizes have indicated a deficiency of Vitamin D in IPF patients, and its potential role in improvement, but the lack of large-scale randomized controlled trials (RCTs) limits definitive conclusions.

Mendelian randomization (MR) is a widely used method for investigating the causal relationship between an exposure and an outcome. By using genetic variants as instrumental variables (IVs), MR helps to mitigate confounding and reverse causation biases that are inherent in observational studies. Thus, an MR study can be thought of as a randomized controlled trial (RCT) that allows for causal inference of the effect of the exposure on the outcome [12]. Although current research suggests a possible association between vitamin D deficiency and IPF, the reliability of existing evidence is low due to the incomparability of study designs and methodological limitations. The aim of this study is to conduct a two-sample MR analysis to determine the potential role of circulating vitamin D status in the risk of IPF.

## Method

### Study design and data source

**Fig. 1:**
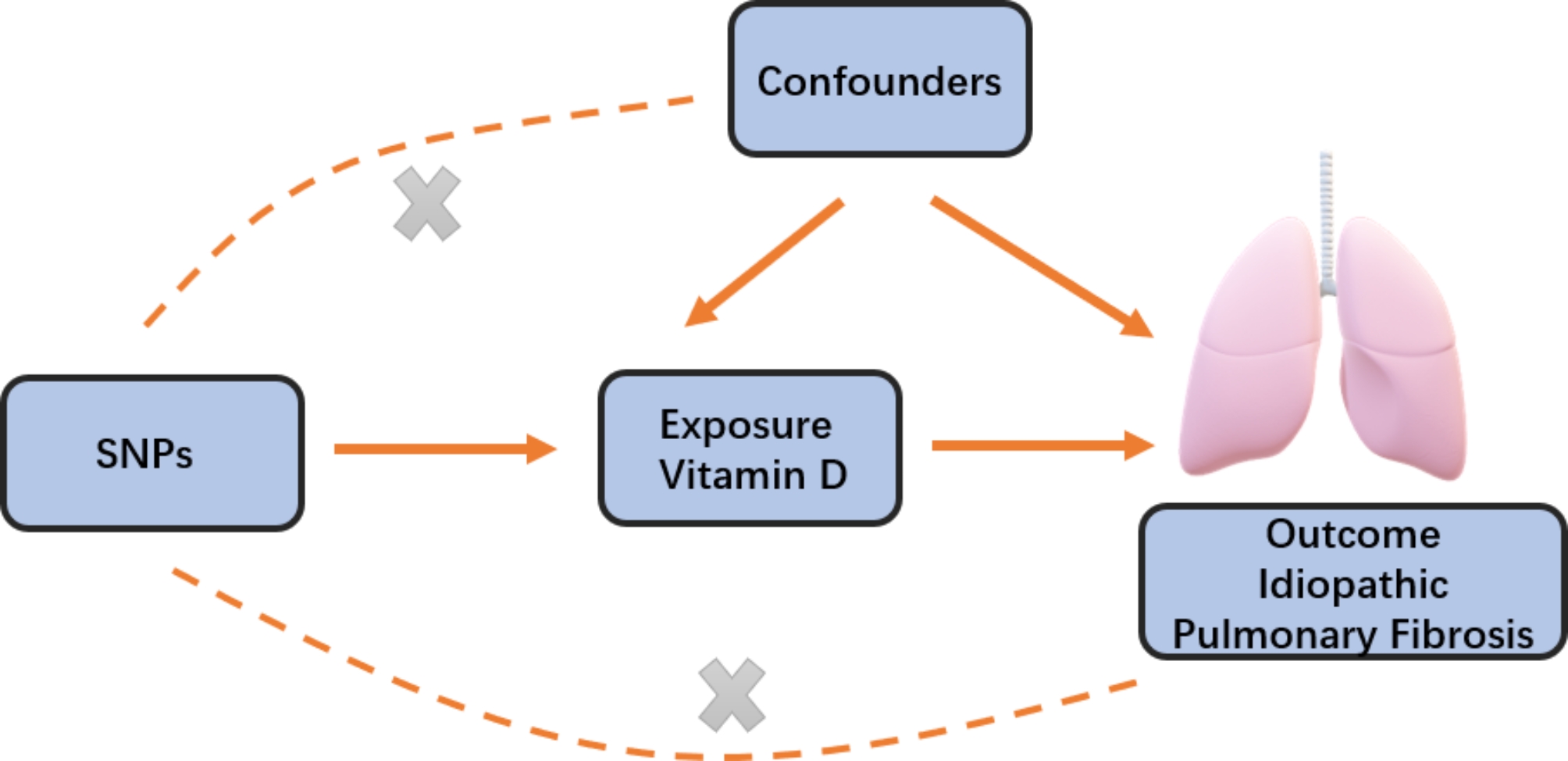
Schematic diagram of the Mendelian randomization assumptions

In order to assess the potential causal relationship between 25(OH)D concentrations and IPF, we conducted a two-sample Mendelian randomization (MR) analysis [13]. The validity of instrumental variables relies on meeting three key assumptions. Firstly, the genetic variants utilized as instrumental variables should be significantly associated with the exposure of interest. Secondly, the genetic variants should not be associated with any confounding factors. Thirdly, the genetic variants should only impact the outcome through the exposure and not through other pathways (Fig. 1). Genetic data on the association between 25(OH)D and IPF were obtained from recently published genome-wide association studies (GWAS). The ethical reviews and informed consent obtained from the original studies were also specific to this research. We utilized single-nucleotide polymorphisms (SNPs) as instrumental variables (IVs) from a genome-wide association study (GWAS) conducted on the UK Biobank (UKB) cohort by Manousaki et al. [14]. A total of 401,460 participants of European descent from the UK Biobank were included in this study, with measurements taken for both 25OHD levels and genome-wide genotypes. Summary statistics for IPF were extracted from the GWAS conducted by Dhindsa et al. [15]. This GWAS analysis included 1028 IPF patients and 196,986 controls.

**Table 1:** Causal effect of circulating Vitamin D levels on IPF

Exposure	Outcome	NO.(SNP)	Method	OR (95% CI)	*P*
25(OH)D	IPF	158	MR Egger	0.984(0.414–2.339)	0.971
25(OH)D	IPF	158	Weighted median	1.504(0.634–3.573)	0.354
25(OH)D	IPF	158	Inverse variance weighted	0.891(0.523–1.518)	0.670
25(OH)D	IPF	158	Simple mode	1.076(0.091–12.746)	0.954
25(OH)D	IPF	158	Weighted mode	1.452(0.670–3.145)	0.346

IPF: idiopathic pulmonary fibrosis; SNP: single-nucleotide polymorphism; OR: odds ratio; CI: confidence interval

**Table 2:** Pleiotropy and heterogeneity tests of MR

Test	Method	Effect size	*P*
Heterogeneity	Q MR Egger	181.320	0.081
	Q IVW	181.415	0.089
Pleiotropy	MR-Egger regression	-0.002	0.774

Q: Cochran’s Q test; MR: Mendelian randomization; IVW: inverse variance weighted

**Fig. 2:**
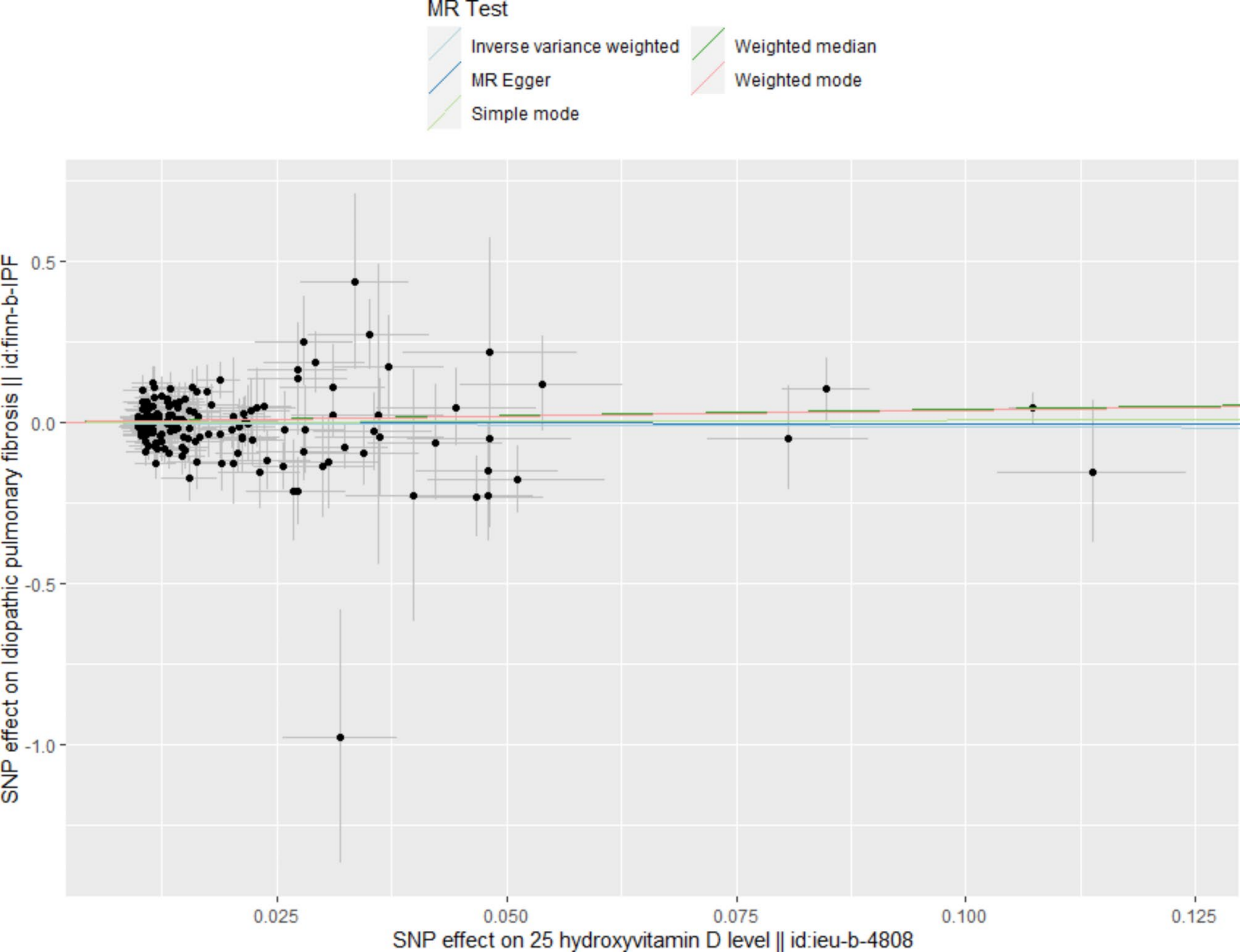
Scatter plot for the causal effect of 25(OH)D levels on IPF risk. The slope of the straight line indicates the magnitude of the causal association

**Fig. 3:**
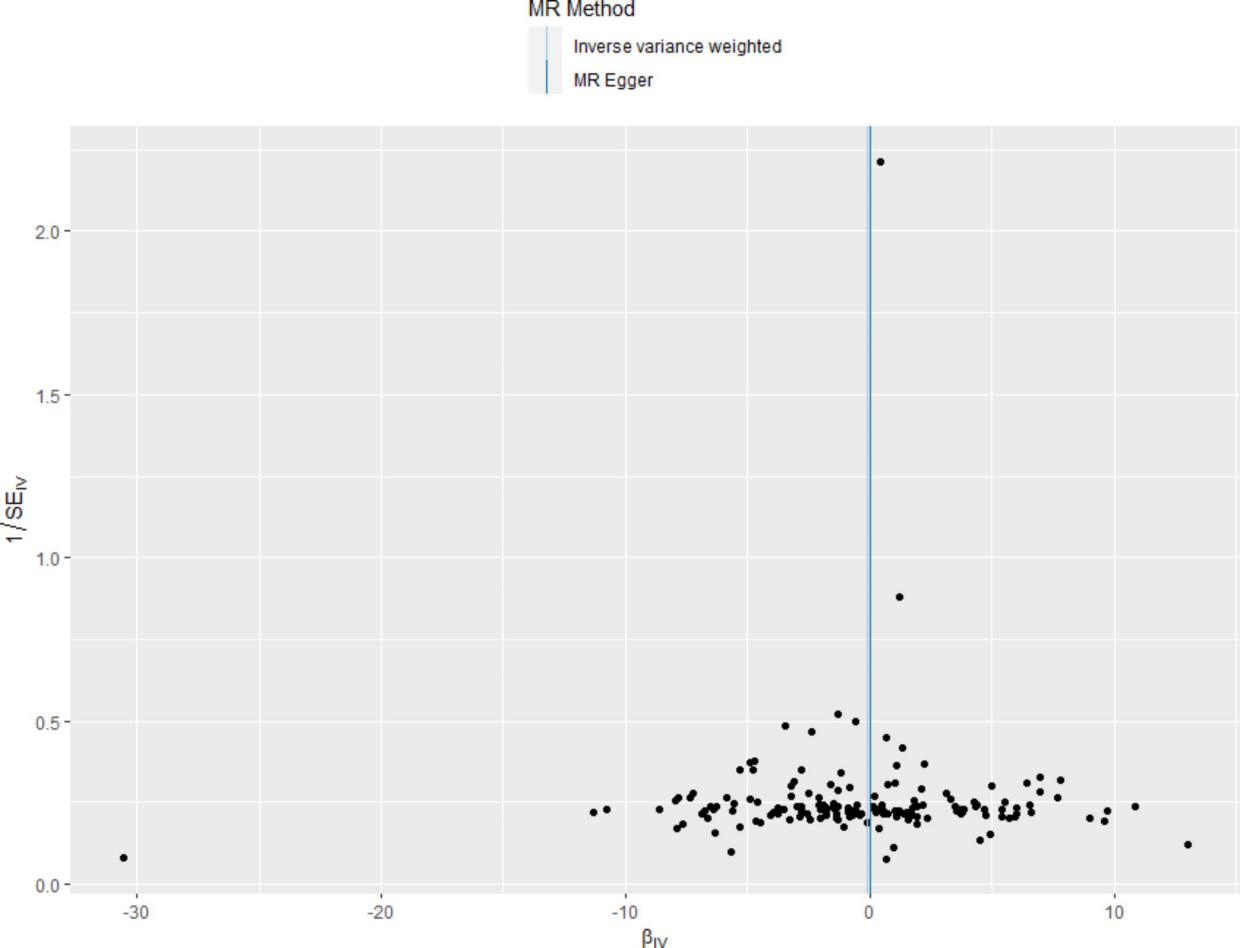
Funnel plot for the overall heterogeneity in the effect of 25(OH)D levels on IPF risk

### Ethical approval

The summary statistics for MR study were obtained from GWAS (https://www.ebi.ac.uk). All these data got ethical approval and freely available.

### Genetic instrumental variables

In order to fulfill the first hypothesis of Mendelian randomization (MR) analysis, which posits that the instrumental variables (IVs) are strongly linked to the 25-hydroxyvitamin D (25OHD) biomarker, we selected independent IVs that were statistically significant in their association with 25OHD at the genome-wide level (P < 5 × 10 − 8, linkage disequilibrium < 0.001, genetic distance = 10,000 KB, minor allele frequency > 0.01).

To avoid potential confounding effects from genetic variants, we conducted a search in the PhenoScanner database[16] (https://www.phenoscanner.medschl.cam.ac.uk) to determine whether the included instrumental variables (IVs) were associated with any known confounding factors. Currently, there are established risk factors for idiopathic pulmonary fibrosis (IPF), such as smoking, exposure to dust and reflux esophagitis [1]. Therefore, in this study, we excluded individuals with these variants were excluded if any association was found. Third, we calculated the F-statistic (F = beta2/se2) [17], since the included IVs were susceptible to weak IVs [18].

### Statistical analysis

To ensure the reliability and validity of our findings, we employed a range of robust statistical methods and conducted sensitivity analyses to assess the potential impact of various sources of bias. The primary analysis was conducted using an inverse-variance weighted (IVW) meta-analysis[19] under a random-effects model. To assess the robustness of the results, we performed four additional sensitivity analyses: the weighted-median method [20], MR-Egger method [21], weighted mode [22] and simple mode [23]. The weighted-median method was employed to obtain valid estimates when more than 50% of information came from valid instrumental variables (IVs). The MR-Egger method was used to evaluate the presence of horizontal pleiotropy among selected IVs [21]. We also assessed heterogeneity among selected IVs using Cochrane’s Q-value [24]. Furthermore, we conducted a leave-one-out sensitivity analysis to examine whether individual single-nucleotide polymorphisms (SNPs) disproportionately affected the overall estimates [25]. We considered suggestive evidence of association when the p-value was between the Bonferroni-corrected value and 0.05, and further confirmation was required. All statistical analyses were conducted using the “TwoSampleMR” packages in R version 4.2.3 (R Foundation for Statistical Computing, Vienna, Austria).

## Results

After the clumping process, we identified 158 SNPs (Additional file 1) as instrumental variables (IVs) to investigate the genetic association between Vitamin D levels and IPF. Removed the following SNPs for being palindromic with intermediate allele frequencies: rs136224, rs2286779, rs2470937, rs2618487, rs589030, rs61826000, rs7660883 and two SNPs related smoking (rs11928368, rs17309874) was excluded as a confounding factor. Then that was displayed in a forest plot (Additional file 2). Subsequently, we conducted Mendelian randomization (MR) analysis using the remaining 158 SNPs, and the results from the IVW method showed no significant causal effect of Vitamin D levels on IPF risk IVW (OR = 0.891, 95% CI 0.523–1.518, *P* = 0.670) (Table 1). Similarly, MR-Egger (OR = 0.984, 95% CI 0.414–2.339, *P* = 0.971), weighted median (OR = 1.504, 95% CI 0.634–3.573, *P* = 0.354), simple mode (OR = 1.076, 95% CI 0.091–12.746, *P* = 0.954) and weighted mode (OR = 1.452, 95% CI 0.670–3.145, *P* = 0.346) (Table 1), all indicated no significant association between Vitamin D level and IPF, as shown in the scatter plot (Fig. 2). Furthermore, we conducted sensitivity analyses to verify the robustness of our findings. Firstly, Cochran’s Q test results showed no heterogeneity among the IVs (*P*_IVW_=0.089, *P*_MR Egger_=0.081, Table 2). The symmetry of the funnel plot also confirmed the absence of heterogeneity (Fig. 3). Secondly, the MR-Egger regression results indicated no overall horizontal pleiotropy across all IVs (*P* = 0.774, Table 2) suggesting that the IVs are unlikely to influence IPF risk through pathways other than Vitamin D levels. Finally, the leave-one-out sensitivity analysis, which involved removing one SNP at a time, yielded consistent results (Additional file 3).

## Discussion

In the current MR analysis, we utilized summary statistics from two GWAS conducted on serum 25(OH)D and IPF in European populations and constructed a strong instrumental variable for 25(OH)D using SNPs. We applied a range of MR methods to investigate the relationship between 25(OH)D and IPF. However, none of these analyses provided evidence of a causal relationship between 25(OH)D concentration in the general population and IPF.

Vitamin D is an essential steroid pro-hormone for the regulation of calcium and phosphorus balance of bone and muscle, but also plays a role in lung tissue remodeling, maintaining lung function and immune system regulation [26]. As it enhances the antimicrobial effects of macrophages and monocytes, promotes chemotaxis and phagocytic capabilities of innate immune cells, and activates the transcription of antimicrobial peptides such as defensin β2 and cathelicidin antimicrobial peptide. Additionally, Vitamin D can induce tolerogenic properties in dendritic cells (DC) to induce a more immature and tolerogenic state, leading to the induction of potential regulatory T cells which are crucial for controlling immune responses and the development of autoreactivity [27]. Vitamin D deficiency concentrations have been linked to elevated mortality caused by severe infections, including upper respiratory tract infections [28], chronic obstructive pulmonary disease [7]. However, clinical trials have shown that Vitamin D does not improve asthma outcomes [29]. Respiratory tract infection is one of the important predisposing factors of acute exacerbation in IPF patients [30]. Although it has been reported Vitamin D supplementation can prevent acute respiratory tract infections [28].

Unfortunately, there is a lack of large-scale clinical trials of IPF and Vitamin D, and several prospective studies have shown a possible association between Vitamin D deficiency and acute exacerbation of IPF. A prospective study by Yang et al. found that decreased serum 25(OH)D was associated with an increased risk of acute exacerbation in patients with IPF including clinical data from 72 patients with IPF (31 stable IPF and 41 acute exacerbation) [10]. In another prospective study of nutritional status assessment in patients with IPF, Vitamin D deficiency was observed in 56.3% of cases [9].

In a recent study, Tzilas et al. observed a deficiency in serum vitamin D concentrations among patients with idiopathic pulmonary fibrosis (IPF) in a clinical sample. This deficiency was found to be closely associated with disease severity and clinical prognostic indicators of respiratory function progression in these patients. Furthermore, they demonstrated through animal experiments inducing lung fibrosis with bleomycin that Vitamin D has a preventive effect against pulmonary fibrosis. Specifically, they observed that pre-treatment with vitamin D significantly reduced the responsiveness of mouse lung fibroblasts (MLFs) to pro-fibrotic stimuli, as indicated by significant reductions in hydroxyproline, collagen, and alpha-smooth muscle actin (α-SMA). This effect was found to be associated with the role of vitamin D in restoring downregulated downregulation of vitamin D-receptor mRNA levels induced by TGFB1 [11]. IPF is generally considered to be caused by persistent stimulation of genetic or environmental factors that damage lung epithelial cells and activate fibroblasts, leading to lung interstitial remodeling [31]. However, the pathogenesis of idiopathic pulmonary fibrosis (IPF) involves a complex interplay of cell types and signaling pathway [32]. Although all these studies have identified vitamin D deficiency in patients with IPF, these observational studies are vulnerable to potential confounding or reverse causality. Moreover, the bleomycin-induced mouse model of pulmonary fibrosis is questionable as a representative of IPF.

Nonetheless, there are some restrictions to the study. Our research was constrained to European ancestry populations. While this may mitigate the bias resulting from population stratification, it remains uncertain whether the outcomes can be extrapolated to other populations. Additionally, we cannot completely disregard the possibility of diet-gene or gene-environment interactions influencing our findings. Our study has several strengths. First, this is the first MR Study to assess Vitamin D levels and IPF risk and that the association is causal. Second, this MR Study was based on two large sample GWAS data from a European population, which provided us with sufficient power to estimate causality. Third, the MR Analysis revealed no causal association of Vitamin D levels with IPF, which is unlikely to be affected by confounding factors.

In addition, the study has several limitations. First, our findings, which are based primarily on participants of European ancestry, may not apply to populations of other ethnic groups. Second, although we did not find the presence of horizontal pleiotropy, there may be residual bias because the exact function of most of these SNPS is unknown. Third, because our study used GWAS summary data rather than individual-level data, we were unable to stratifying our analyses according to other factors such as age and sex. Fourth, as epidemics and diagnostic technologies advance, the diagnosis of IPF is likely to experience transformations. Currently, in the UK Biobank dataset, IPF diagnosis relies solely on diagnostic codes. However, this approach may lack the necessary specificity for accurately identifying IPF and could inadvertently encompass other Interstitial Lung Diseases (ILD) that have distinct pathophysiological mechanisms. The presence of these diverse ILDs within the dataset may lead to variations in associations with vitamin D levels, potentially influencing the outcomes of our study.

## Conclusions

In general, our research indicates that there is no significant association between Vitamin D levels and the development of IPF. However, the exact mechanisms between Vitamin D and IPF remain unclear, and further pathological and biochemical studies are needed to explore this issue.

### Electronic supplementary material

Below is the link to the electronic supplementary material.

Additional file 1: IVs for circulating 25(OH)D levels.

Additional file 2: The 158 SNPs IVs between Vitamin D levels and IPF displayed in a forest plot.

Additional file 3: The 158 SNPs leave-one-out sensitivity analysis.

## Data Availability

All data generated or analysed during this study are included in this published article and its supplementary information files.
