# Supplementary material for: Vitamin D and idiopathic pulmonary fibrosis: a two-sample mendelian randomization study
Source: BMC Pulm Med. 2023 Aug 23;23:309. doi: 10.1186/s12890-023-02589-z (PMC10463904; doi:10.1186/s12890-023-02589-z)
Supplement: Supplementary file 2 — Additional file 2: The 158 SNPs IVs between Vitamin D levels and IPF displayed in a forest plot. [file 12890_2023_2589_MOESM2_ESM.pdf]

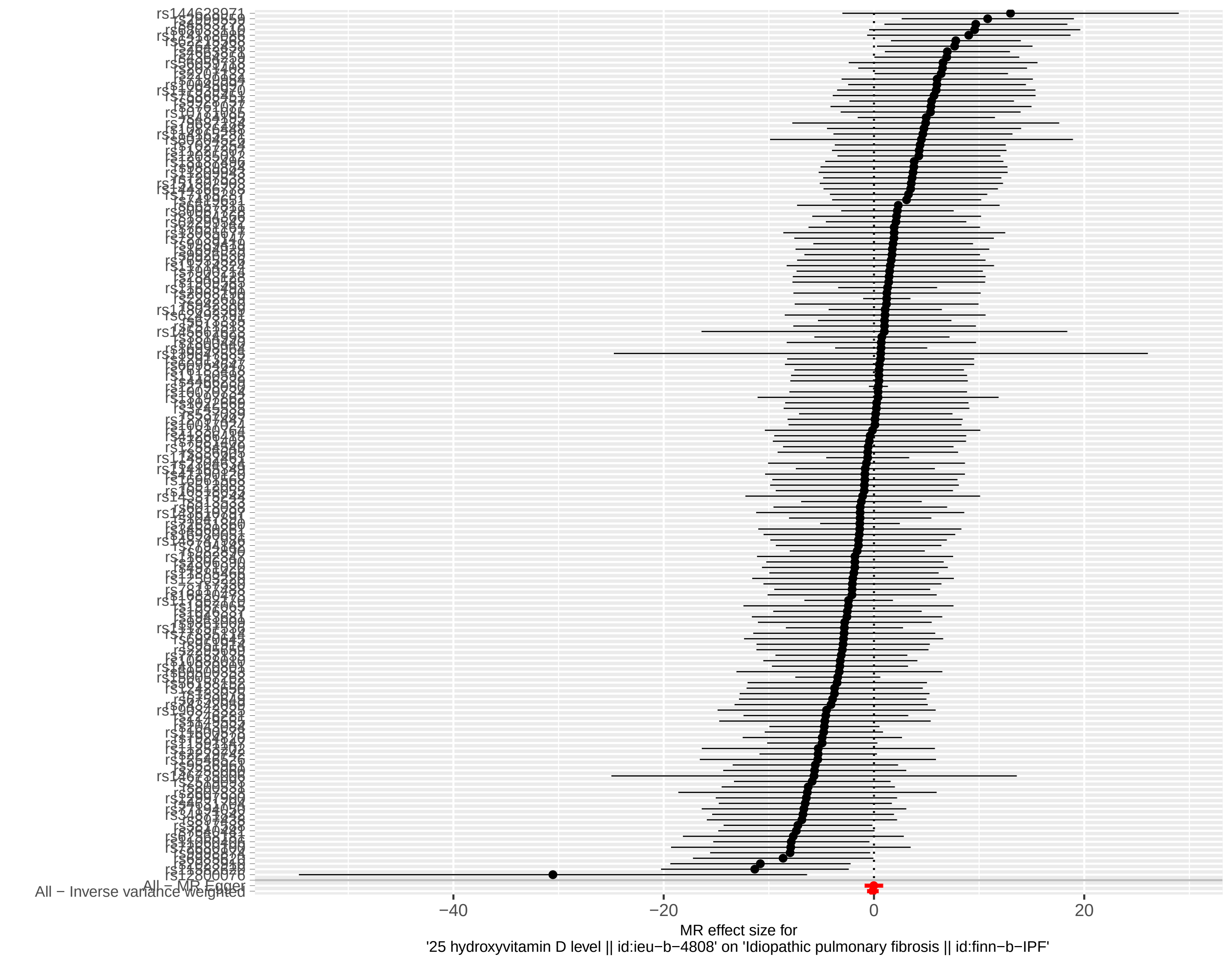
rs144628971
rs12800076
All − MR Egger
All − Inverse variance weighted
−40
−20
0
20
MR effect size for
'25 hydroxyvitamin D level || id:ieu−b−4808' on 'Idiopathic pulmonary fibrosis || id:finn−b−IPF'
