# Supplementary figures and images for: Vitamin D and idiopathic pulmonary fibrosis: a two-sample mendelian randomization study
Source: BMC Pulm Med. 2023 Aug 23;23:309. doi: 10.1186/s12890-023-02589-z (PMC10463904; doi:10.1186/s12890-023-02589-z)

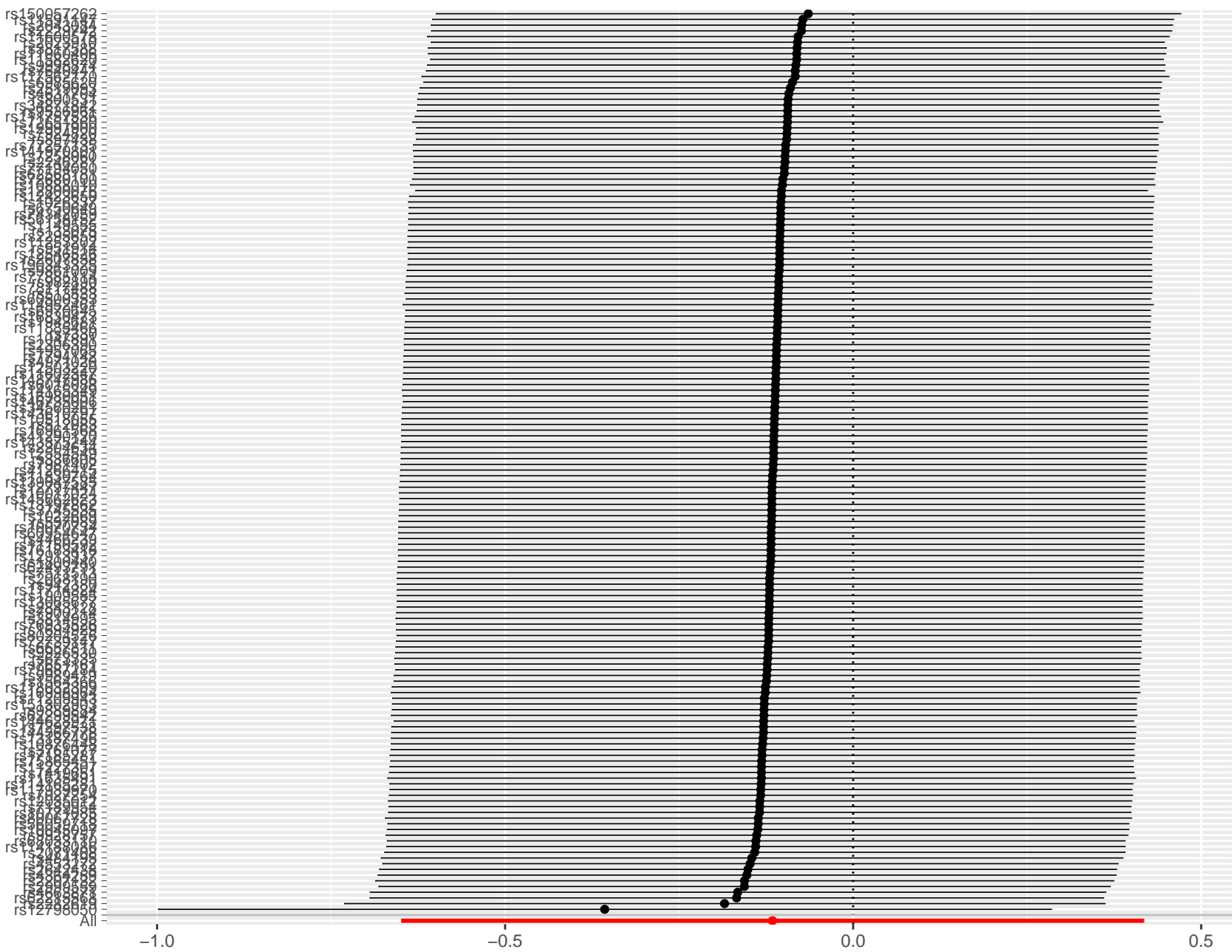

Supplement: Supplementary file 3 — Additional file 3: The 158 SNPs leave-one-out sensitivity analysis. [file 12890_2023_2589_MOESM3_ESM.pdf]
